# Organophosphate Nerve Agent Detection with Europium Complexes

**DOI:** 10.1100/tsw.2004.194

**Published:** 2004-11-05

**Authors:** Jake R. Schwierking, Laird W. Menzel, E. Roland Menzel

**Affiliations:** Center for Forensic Studies, Physics Department, Texas Tech University, Lubbock, TX 79409-1051, USA

**Keywords:** organophosphate nerve agents, paraoxon, diisopropylfluorophosphate, photoluminescence, europium, sensitizing ligands, 1, thenoyltrifluoroacetone

## Abstract

We explore the detection of paraoxon, a model compound for nonvolatile organophosphate nerve agents such as VX. The detection utilizes europium complexes with 1,10 phenanthroline and thenoyltrifluoroacetone as sensitizing ligands. Both europium luminescence quenching and luminescence enhancement modalities are involved in the detection, which is simple, rapid, and sensitive. It is adaptable as well to the more volatile fluorophosphate nerve agents. It involves nothing more than visual luminescence observation under sample illumination by an ordinary hand-held ultraviolet lamp.

## INTRODUCTION

In previous articles, we described a field method for the detection of traces of explosives. One of the detection modalities involved tagging with certain lanthanide complexes[1,2]. Similarly, such complexes were investigated for purposes of field-detecting volatile fluorophosphate nerve agents in vapor form, utilizing diisopropylfluorophosphate as the model compound[3]. In this article, we explore the use of such lanthanide complexes to detect nonfluorine-containing nerve agents that are not volatile. To set the stage for this investigation, we first review the chemical mechanisms involved in the explosives and fluorophosphate cases. They are pertinent to the present study, which aims at simple, rapid, and sensitive field methodology.

The normal photoluminescence of the trivalent lanthanide ions can be massively increased when certain organic ligands bind to the lanthanide. These ligands, referred to as sensitizing ligands, absorb incident (ultraviolet) light. The excitation energy is efficiently transferred to the lanthanide ion, causing it to luminesce much more intensely than what is obtained by direct optical excitation of the lanthanide ion. Awareness of this sensitization dates back to WW II[4]. Mostly, europium and terbium complexes are employed. They are the strongest lanthanide luminescers. Europium is of special interest because it is compatible with a greater range of sensitizing ligands than terbium. Thus, we focus on europium complexes in the present study. Effective sensitizing ligands are 1,10 phenanthroline (ortho-phenanthroline, OP) and thenoyltrifluoroacetone (TTFA). Both are compatible with near-ultraviolet excitation. OP is also compatible with deep-ultraviolet excitation. Both ligands are bidentate. Fig. 1 shows the binding of PO and TTFA to the europium. Given that the coordination number of $Eu^{3+}$ is nine, four such ligands can bind to the europium. The ninth binding site is usually occupied by a water of hydration. Typically, the complexes are prepared by mixing $EuCl_3 \cdot 6H_2O$ with the ligands in a polar solvent. The reaction is essentially instantaneous.

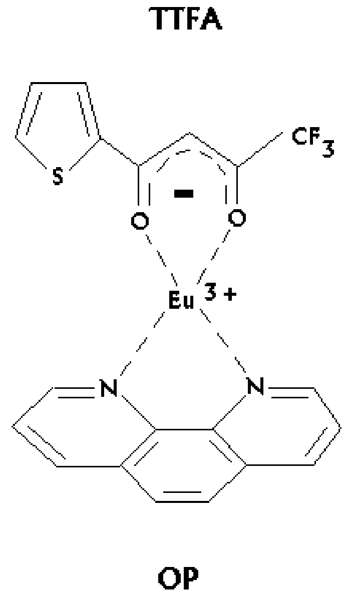

**FIGURE 1.** Binding of OP and TTFA to europium.

Water is known to be a quencher of lanthanide luminescence[5]. One water bound to the europium complex is still tolerable, but additional water binding (by replacing one or more of the sensitizing ligands) strongly quenches the europium luminescence. Similarly, presence of excess interstitial water molecules that loiter in the vicinity of the europium complex will cause strong luminescence quenching. This effect of water is taken advantage of in the detection of explosives. The sensor is a piece of filter paper with which the explosive-contaminated article is swiped. Alternatively, the explosive, dissolved in acetone, is spotted on the filter paper. The filter paper is next immersed in a (methanol) solution of the Eu-OP or -TTFA complex. The complex binds to the $NO_2$ functionality of the explosive, whereby the water occupying the ninth binding site is displaced. This produces a hydrophobic environment around the europium ion, such that luminescence quenching by water is inhibited. A subsequent soaking of the filter paper in water quenches the luminescence of the unreacted europium complex, but not that of the complex attached to the explosive.

The sensor for the detection of fluorophosphate vapor is a piece of filter paper impregnated with a methanol solution of Eu-TTFA and left to dry. Methanol is a hygroscopic solvent, and water molecules are present in the paper in any event because europium chloride hexahydrate is used in the complex preparation. The incident fluorophosphate hydrolizes very quickly to form HF. The very aggressive fluoride anion displaces the negatively charged TTFA ligand and, consequently, also allows added hydration. Thus, luminescence sensitization is diminished and luminescence quenching is increased, producing strong europium luminescence quenching. This quenching is very quickly realized. Subsequently, the phosphate product of the fluorophosphate hydrolysis can also bind to the Eu-TTFA complex, to displace water of hydration. This is a slow process because the sensor is a dry solid and the fluorophosphate is applied in vapor form. The fluoride anion eventually solvates in water to lose its potency, such that the TTFA ligand can rebind to europium. The upshot is an eventual recovery of the europium luminescence. As we shall see, phosphate binding in solution is very much more rapid, essentially instantaneous. Eu-OP is not effective in the above manner because OP is a strong ligand and acts as a neutral ligand. It thus is resistant to attack by the fluoride anion.

## DETECTION OF NONVOLATILE ORGANOPHOSPHATES

Fluorophosphate nerve agents such as Sarin have considerable volatility and thus can be deployed in vapor form. Other organophosphate nerve agents, such as the very lethal VX, which does not contain F, are not volatile and thus are deployed in liquid form, as aerosol (perhaps via munition explosion) or spray (akin to crop dusting). The vapor pressure of Sarin, for instance, is about 3 mmHg (at room temperature), whereas that of VX is about $10^{-3}$ mmHg[6]. In our study, we use the model compound paraoxon (-ethyl), diethyl-p-nitrophenyl phosphate, which is frequently used for this purpose[7,8], just as diisopropylfluorophosphate (vapor pressure about 0.6 mmHg) frequently serves as the model compound for Sarin. Paraoxon is a potent pesticide. Its vapor pressure is about $10^{-5}$ mmHg. Fig. 2 shows the structures of VX, paraoxon, and the products of paraoxon hydrolysis. The model compounds are not nearly as lethal as the corresponding nerve agents, but, like the nerve agents, they inhibit acetylcholinesterase (leading to paralysis and death).

### Sensor Preparation

The Eu-TTFA and -OP complexes are prepared by simply mixing in methanol the $EuCl_3 \cdot 6H_2O$ with TTFA or OP. We use a 1:4 molar ratio of Eu:ligand and a $10^{-2}$ *M* complex concentration. For reasons discussed below, it is useful to have the complex dissolved in a nonpolar solvent, heptane in our work. To achieve this, we simply add heptane to the methanol solution, in volume equal to the methanol. The mixture is then stirred and sonicated for 5–10 min. Eu-TTFA readily migrates into the heptane fraction. On standing, the mixture separates, with the heptane fraction on top of the methanol fraction. Illumination of the sample with near-ultraviolet light from a hand-held ultraviolet lamp (Model UVGL-58, Mineralite, UVP, Upland, CA) reveals the characteristic red europium luminescence in both solvent fractions. On the basis of the luminescence intensities, we estimate the relative complex concentrations to be 3:2 in the methanol:heptane fractions, i.e., $6 \times 10^{-3}$ *M*:$4 \times 10^{-3}$ *M*. Eu-OP does not easily migrate from methanol to heptane. The relative concentrations in our preparation are roughly 10:1 in methanol:heptane. Our sensor is simply either the methanol or heptane fraction thus prepared. For the sensing of the paraoxon model compound, the sensor solution is in 50-drop volume (about 1 ml). In some experiments, the effect of hydrolysis is assessed. In those instances, two drops of water are added to the 50-drop solution. One (undiluted) drop of the model compound is then added to each solution. The solution is then agitated (shaken) for a few seconds. All reagents, including the paraoxon, were obtained from Sigma-Aldrich and were used as received.

### Results

#### *Eu-OP*

In all instances, the introduction of the paraoxon drop produces strong europium luminescence quenching. Paraoxon is a yellow, somewhat oily, liquid that dissolves in both methanol and heptane. In the heptane/water case, the agitation temporarily forms an emulsion. The water interacts with the paraoxon to quickly form the hydrolysis products. On subsequent standing, the mixture separates, with the water

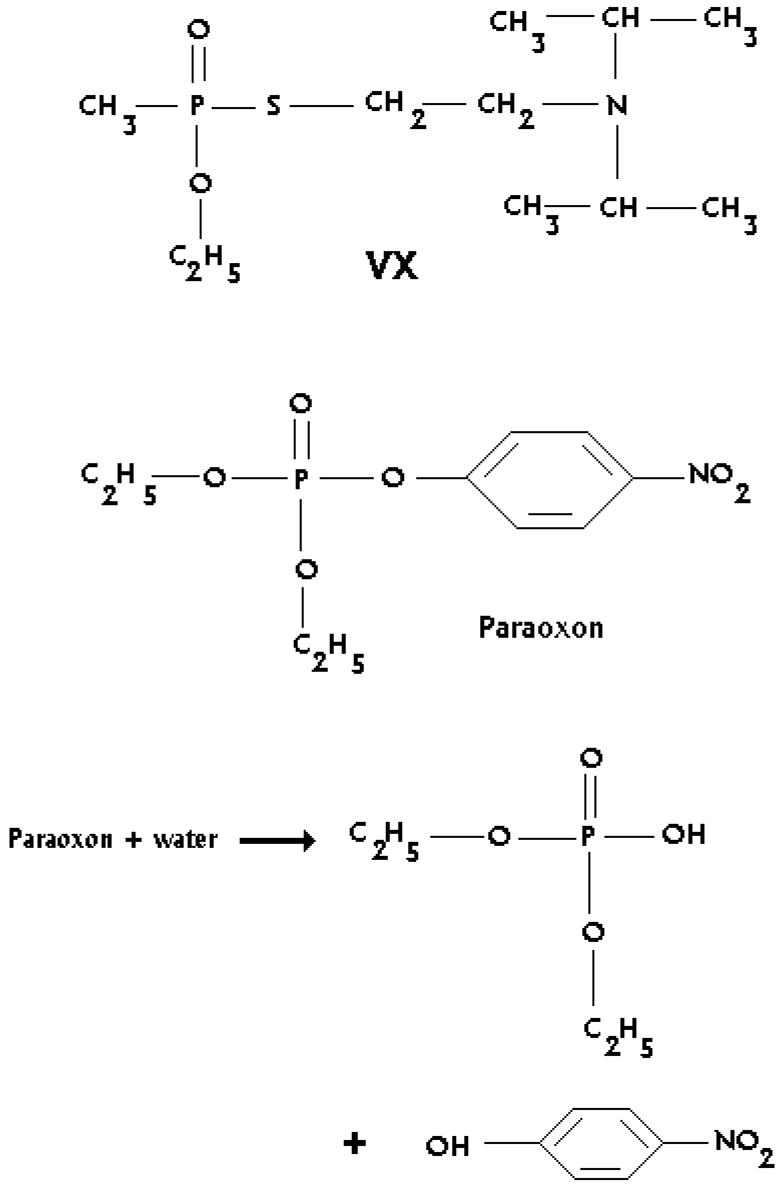

**FIGURE 2**. Structures of VX and paraoxon, and products of paraoxon hydrolysis.

fraction below the heptane fraction. Fig. 3 shows five sample vials in room light. The left-most vial contains the Eu-OP in methanol/water control. In order from left to right, the other four vial solutions are methanol, methanol/water, heptane, and heptane/water, with one drop of paraoxon added to each of these four vials (and followed by agitation). From left to right, the colors of the solutions were clear, yellow, yellow, faint yellow, and clear. Comparison of the latter two vials indicates the occurrence of hydrolysis. Our digital camera is an antiquated one not suitable for close-up work. Thus, Fig. 3 and subsequent ones are not in sharp focus. This is not important, however, in that the essential content is color and intensity, which is reproduced in the photos. Fig. 4 is the luminescence photograph corresponding to Fig. 3. Only the control shows luminescence. It is totally quenched in the other four vials.

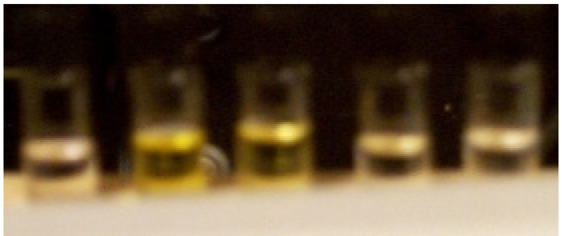

**FIGURE 3.** Room light photo of Eu-OP samples. See text. Left to right in: methanol/water, clear; methanol + 1 drop of paraoxon, yellow; methanol/water + 1 drop of paraoxon, yellow; heptane + 1 drop of paraoxon, faint yellow; heptane/water + 1 drop of paraoxon, clear.

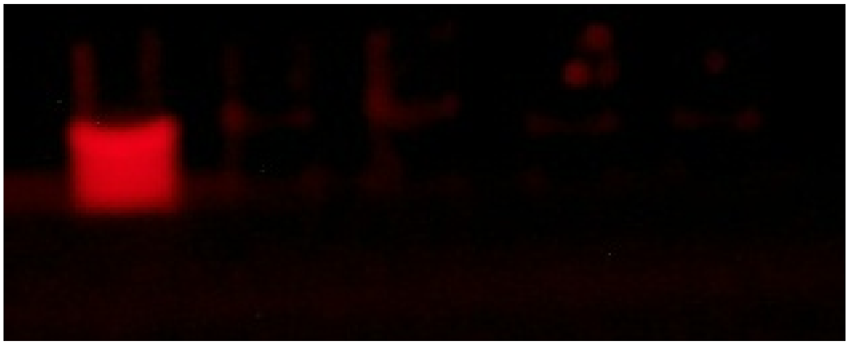

**FIGURE 4.** Luminescence photo of samples of Fig. 3.

### *Eu-TTFA*

In room light, methanol, methanol/water, heptane, and heptane/water solutions (1 ml) to which one drop of paraoxon is added are yellow, yellow, faint yellow, and clear, respectively. We find that the addition of the paraoxon has no effect to speak of on the europium luminescence in the methanol, methanol/water and heptane solutions. However, the europium luminescence strongly increases in the heptane/water solution.

## Sensitivity

To assess the sensitivities of the above modalities, Eu-OP quenching and Eu-TTFA enhancement in heptane/water, one drop of paraoxon was diluted with nine drops of either methanol or heptane to produce dilution by a factor 10. One drop of this dilution was next mixed with nine drops of solvent to produce dilution by a factor 100. Etc. One drop of each dilution was then added to the sensor solutions to assess sensitivity. For both the Eu-OP quenching and the Eu-TTFA enhancement, the sensitivity limit was dilution by a factor 100. This is shown for Eu-TTFA in the luminescence photograph of Fig. 5. From left to right, the vials contain Eu-TTFA in methanol/water, methanol, heptane/water, heptane/water, heptane/water, and heptane/water, with one drop of undiluted paraoxon added to the two left-most and the right-most vials, one drop of paraoxon dilution by 10 and 100 in the second- and third-from-right vials, respectively, and no paraoxon in the fourth-from-right vial, which thus serves as control. The europium luminescences from the two left-most vials, compared with the control, show that the paraoxon, hydrolized or not, has no significant effect. For Eu-TTFA in heptane without water, the observed luminescence on addition of one undiluted drop of paraoxon (not shown in the figure) is equal to that of the left-most vial.

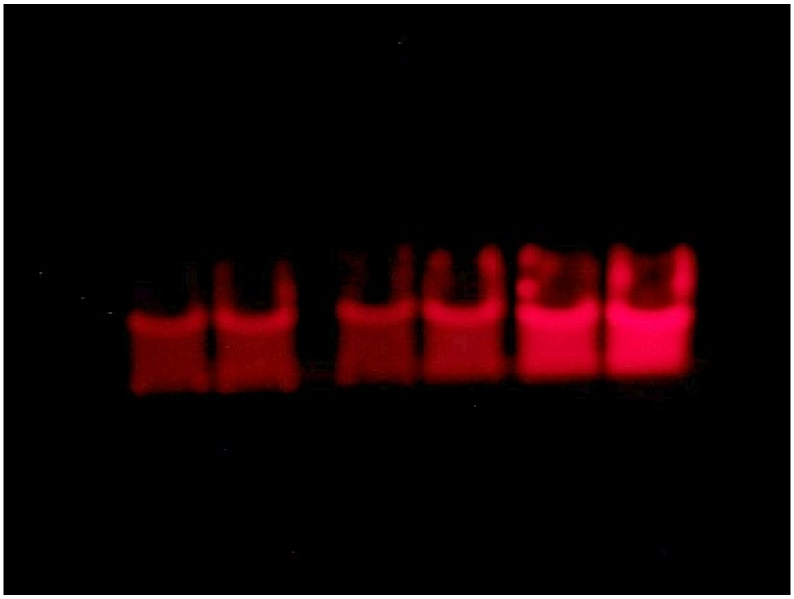

**FIGURE 5**. Luminescence photo of Eu-TTFA samples. See text. Left to right in: methanol/water + 1 drop of undiluted paraoxon; methanol + 1 drop of undiluted paraoxon; heptane/water; heptane/water + 1 drop of by 100 diluted paraoxon; heptane/water + 1 drop of by 10 diluted paraoxon; heptane/water + 1 drop of undiluted paraoxon.

## Phosphate vs. Nitrophenol Hydrolysis Product

In terms of the phosphate functionality, paraoxon does serve as a model compound for organophosphate nerve agents. However, such agents do not contain the nitro functionality of paraoxon. It thus becomes necessary to ensure that paraoxon sensing does not produce false effects due to the nitro functionality. This is a concern in that we know from our earlier work with explosives that the nitro functionality does have the ability to bind to europium complexes. Indeed, when a bit of RDX (which contains three nitro groups) is added to Eu-TTFA in heptane/water, a significant increase in europium luminescence is found, albeit not as pronounced as that obtained with paraoxon. It thus becomes essential to investigate the binding of phosphate to Eu-TTFA. To assess this, four drops of water were mixed with two drops of undiluted diisopropylfluorophosphate. The hydrolysis produces HF and a phosphate very similar to the phosphate hydrolysis product of paraoxon. The two differ only in the substitution of the two ethyl moieties of the latter by isopropyl moieties in the former. To the fluorophosphate/water mix, 20 drops of heptane were next added, followed by agitation for a few seconds. On standing, the mixture soon separated, with the water fraction below the heptane fraction. The objective of the preparation was to have the HF in the water fraction and (at least) a substantial amount of the phosphate hydrolysis product in the heptane fraction. Thus, when the heptane fraction is siphoned off for further use, one does not have to contend with the aggressive HF, but one does have substantial quantities of the phosphate product in the heptane fraction. A few drops of this fraction were then added to about 2 ml of $5 \times 10^{-3}$ $M$ Eu-TTFA in roughly 1:1 methanol:heptane, followed by agitation for a few seconds. On letting the mixture separate, a significantly stronger europium luminescence was found in the supernatant heptane fraction, compared to the methanol fraction. Finally, four drops of water were added, followed again by agitation. On standing, the luminescence result of Fig. 6 was obtained. It shows strong europium luminescence quenching in the methanol fraction and strong enhancement in the heptane fraction. The involved mechanisms are much like those discussed earlier in connection with explosives, with the additional factor that the heptane solvent is very effective in excluding water that is not directly bound to the europium complex. That potentially bound water (ninth binding site) is displaced by the phosphate.

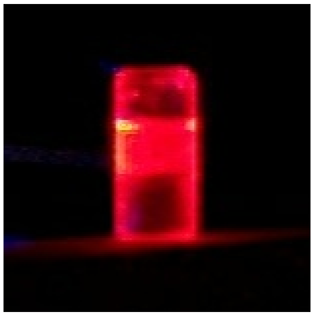

**FIGURE 6**. Luminescence photo showing phosphate binding to Eu-TTFA complex. See text.

## OTHER FIELD METHODS FOR NERVE AGENT DETECTION

Various instrumental methods exist for nerve agent detection, such as gas chromatography/mass spectroscopy, ion mobility, flame spectrometry, etc. Some of them are portable, field-worth, but are expensive. We only know of three sensing approaches that resemble what we describe. One of them involves paper sensors that yield colored products on reaction with nerve agents[9]. We have no information on their speed or sensitivity. A bioassay-type method involving fluorescence is under study[7]. It is based directly on the mechanism of toxicity of the nerve agent. For fluorophosphate nerve agents, it uses a cholinesterase. The liberated HF reacts with a nonfluorescent coumarin derivative to render it fluorescent. The assay, designed for detection of nerve agent prior to release, is reported to take 1–2 min. Our approach is designed for detection after release. A method that involves lanthanide chelates with sensitizing ligands and also the analyte attached during sensor preparation has been reported recently[10]. Once that complex is immobilized in a polymer matrix, the polymer is processed such that the analyte is removed, leaving behind in the polymer a cavity of the dimensions of the analyte. The polymer + cavity system is the sensor, which thus has chemical specificity. When it is subsequently exposed to the analyte, the cavity is reoccupied. This changes the ligand electric field at the site of the lanthanide ion to produce subtle, but measurable, spectral changes in luminescence wavelength and quantum efficiency. The sensing is thus spectroscopy-intensive (and not trivial). We glean from ref. 10 that the sensor response is not fast.

## DISCUSSION

The results with Eu-OP indicate that hydrolysis is not the pertinent issue. The phosphate moiety interacts with the OP-sensitizing ligand to inhibit the energy transfer. This does not involve displacement of the sensitizing ligand, as indicated by the absence of the characteristic intense blue fluorescence of free OP in any of our samples. We do not at present understand the mechanism for the sensitization inhibition. The observation that the paraoxon has no significant effect on Eu-TTFA in methanol or methanol/water indicates that the paraoxon or its hydrolysis products do not displace TTFA ligand or inhibit its sensitization activity. The result of Fig. 6 implies adaptability to fluorophosphate detection.

One can envision numerous false effects that produce europium luminescence quenching. Europium luminescence enhancement is less prone to false effects. We envision a three-compartment sensor that shows quenching in one compartment, enhancement in another, and no effect (control) in the third, to minimize false positives. In principle, it would be advantageous to have a solid, dry sensor, such as the filter paper discussed in connection with fluorophosphate vapor detection. However, sequential spottings would now be involved, and these produce irregular spots with increasing size, including migration of fluorophore in the manner of paper chromatography. We have carried out such spottings. Results are qualitatively similar to what we find in solution, but not as clear and unambiguous.

As little as one drop of VX on the skin can be fatal[11]. Our results suggest that our detection concept should be sensitive enough for effective field use if the paraoxon and diisopropyfluorophosphate findings extrapolate to the actual organophosphate nerve agents. The sensing is quick and simple.

## BIOSKETCH

E. Roland Menzel is Professor of Physics and Chemistry, and is Director at the Center for Forensic Studies Texas Tech University, Lubbock, TX USA. He received a B.S. in 1967 and a Ph.D. in 1970, in Physics at Washington State University. He served postdoctoral fellowships at Simon Fraser University in British Columbia, Purdue University, and the University of Kentucky. His current research involves the application of photoluminescence techniques to trace evidence analysis, primarily fingerprint detection, document examination, and field methods for detection of traces of explosives and organophosphate nerve agents. Professor Menzel received the Lewis Minshall Award in 1995 from The Fingerprint Society, UK and became Honorary Professor, 1995, of the Liaoning Scientific & Technical Research Institute (China). Fingerprint detection with lasers was named a milestone of 20th century Canadian chemistry.
